# BEAVR: a browser-based tool for the exploration and visualization of RNA-seq data

**DOI:** 10.1186/s12859-020-03549-8

**Published:** 2020-05-29

**Authors:** Pirunthan Perampalam, Frederick A. Dick

**Affiliations:** 1 London Regional Cancer Program, London Health Sciences Centre, London, ON N6A 5W9 Canada; 2 Department of Biochemistry, University of Western Ontario, London, ON N6A 5C1 Canada; 3 Department of Pathology and Laboratory Medicine, University of Western Ontario, London, ON N6A 5C1 Canada; 4 Children’s Health Research Institute, London, ON N6A 4V2 Canada

**Keywords:** Data visualization, Data exploration, Principle component analysis, Hierarchical gene clustering, Pathway analysis

## Abstract

**Background:**

The use of RNA-sequencing (RNA-seq) in molecular biology research and clinical settings has increased significantly over the past decade. Despite its widespread adoption, there is a lack of simple and interactive tools to analyze and explore RNA-seq data. Many established tools require programming or Unix/Bash knowledge to analyze and visualize results. This requirement presents a significant barrier for many researchers to efficiently analyze and present RNA-seq data.

**Results:**

Here we present BEAVR, a **B**rowser-based tool for the **E**xploration **A**nd **V**isualization of **R**NA-seq data. BEAVR is an easy-to-use tool that facilitates interactive analysis and exploration of RNA-seq data. BEAVR is developed in R and uses DESeq2 as its engine for differential gene expression (DGE) analysis, but assumes users have no prior knowledge of R or DESeq2. BEAVR allows researchers to easily obtain a table of differentially-expressed genes with statistical testing and then visualize the results in a series of graphs, plots and heatmaps. Users are able to customize many parameters for statistical testing, dealing with variance, clustering methods and pathway analysis to generate high quality figures.

**Conclusion:**

BEAVR simplifies analysis for novice users but also streamlines the RNA-seq analysis process for experts by automating several steps. BEAVR and its documentation can be found on GitHub at https://github.com/developerpiru/BEAVR. BEAVR is available as a Docker container at https://hub.docker.com/r/pirunthan/beavr.

## Background

RNA-sequencing (RNA-seq) has revolutionized molecular biology research in the last decade [1]. RNA-seq is a high-throughput sequencing method that allows for the quantification of gene expression patterns between experimental groups using differential gene expression (DGE) methods [2]. Analysis of DGE may guide the early phases of studies by highlighting transcripts and/or pathways with altered expression in a given experimental system or may be used to assess the downstream impacts of a treatment or other experimental condition. RNA-seq experiments may follow almost any variation of in vitro or in vivo study in which RNA is collected [3]. Most recently, RNA-seq has been employed clinically, including in numerous cancer-related clinical trials [4–6].

Once the wet lab components of an RNA-seq experiment are completed, the data must be analyzed computationally. To date, a multitude of tools are available to researchers depending on the experimental question (e.g. the discovery of novel transcripts or determining gene expression changes) [3, 7]. Regardless of the analysis tool selected, the vast majority of currently available tools require knowledge of programming (C/C++, Perl, Python, R) or shell scripting (Unix/Bash shell). DESeq2, one of the most popular analytical software packages for DGE, is written in R and requires an understanding of this language to manipulate data and visualize results [8]. The requirement for users to navigate one or more computational languages in order to analyze RNA-seq data presents a substantial barrier for many researchers who are adept with respect to the wet lab components of RNA-seq but unfamiliar with the computational aspects.

Here, we present BEAVR, a **B**rowser-based tool for the **E**xploration **A**nd **V**isualization of **R**NA-seq data. BEAVR is an operating system (OS)-independent software package written in R that can run locally on a user’s computer or on a remote server. BEAVR provides an easy-to-use graphical frontend to allow both novices and experts to perform DGE analyses on RNA-seq datasets. Specifically, BEAVR simplifies the process of visualization and exploration of results and allows users to generate visually-appealing graphs, tables, plots, heatmaps and pathways maps. At its core, BEAVR uses the heavily-cited DESeq2 as the engine for its analysis. While there is no single superior method for RNA-seq analyses, DESeq2 is an ideal choice because it requires only raw, unnormalized read counts and provides functions to perform DGE and statistical analyses. Our implementation allows for the visualization of PCA plots, read count plots, volcano plots, heatmaps and enriched pathways and facilitates the exploration of DGE results to aid researchers in their study of known gene interactions as well as providing tools for the discovery of novel gene interactions.

## Implementation

### Interface & typical workflow

**Fig. 1:**
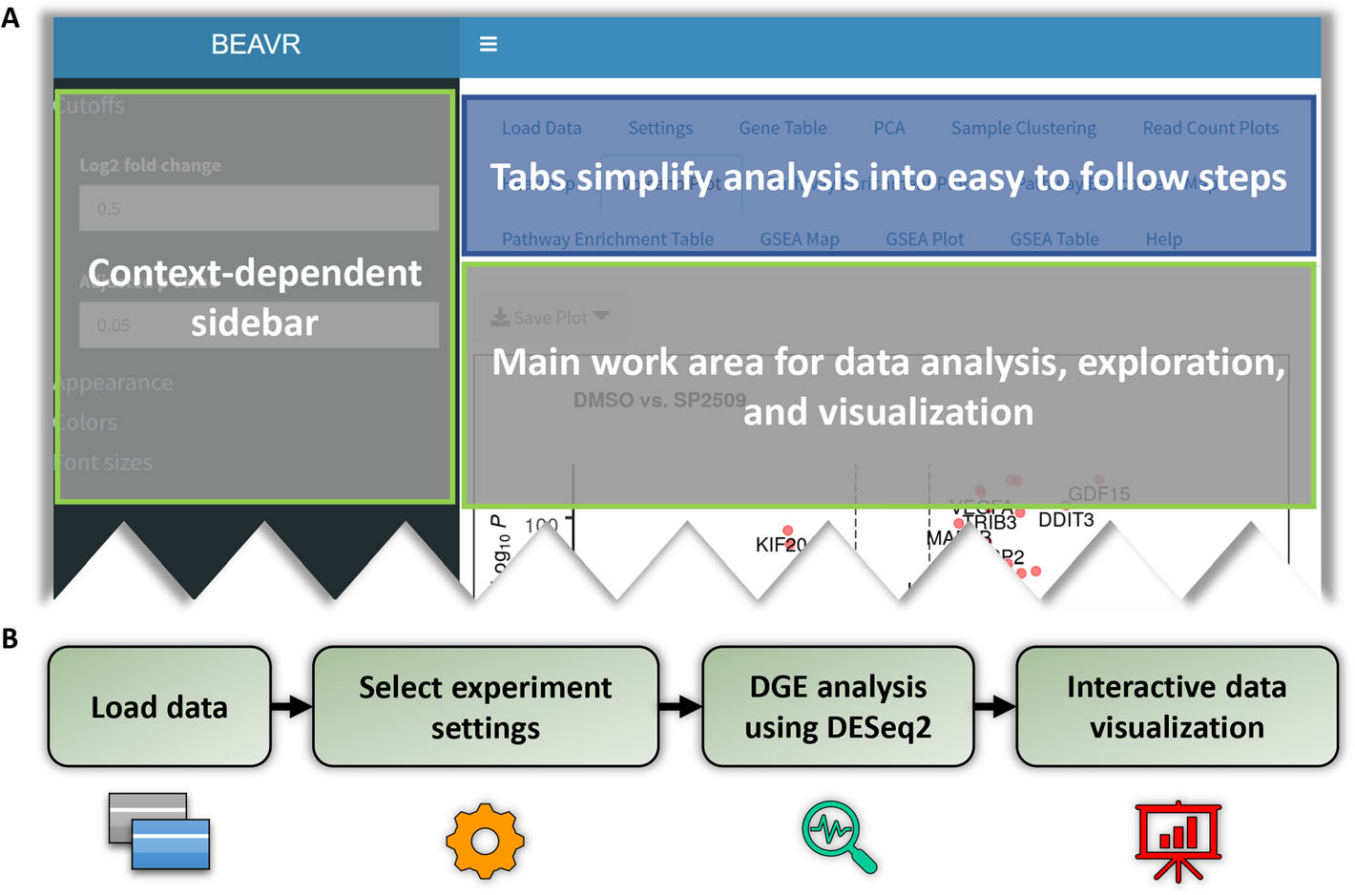
Overview of BEAVR’s graphical user interface and typical workflow. **a**BEAVR’s easy-to-use graphical user interface (GUI) is divided into two areas; a main work area and a sidebar. The main work area has a tabbed-interface to select data output and figure displays. Depending on the tab selected in the main working area, the context-dependent sidebar will show appropriate options and parameters that allow the user to customize analysis, data output and figures. **b**BEAVR breaks down the RNA-seq analysis workflow into logical steps. Users begin by loading their data (raw read counts and sample information) and select experimental settings for analysis and statistical tests. Then differential gene expression (DGE) analysis is performed automatically using DESeq2, lastly the data is displayed in interactive tables, graphs and plots that users can explore, manipulate and customize

BEAVR’s graphical user interface (GUI) is developed in R using the shiny framework. The layout is divided into a main panel and a sidebar panel (Fig. 1a). The main panel presents the user with a tabbed environment that breaks the workflow of DGE analysis into easy-to-follow logical steps. Depending on which tab is open, the sidebar will display context-dependent parameters that control the output and display of data in the work area of the main panel. The user can manipulate these parameters at any time and the results will be recalculated and updated in real-time, drastically reducing the amount of time required compared to command-line based approaches.

**Fig. 2:**
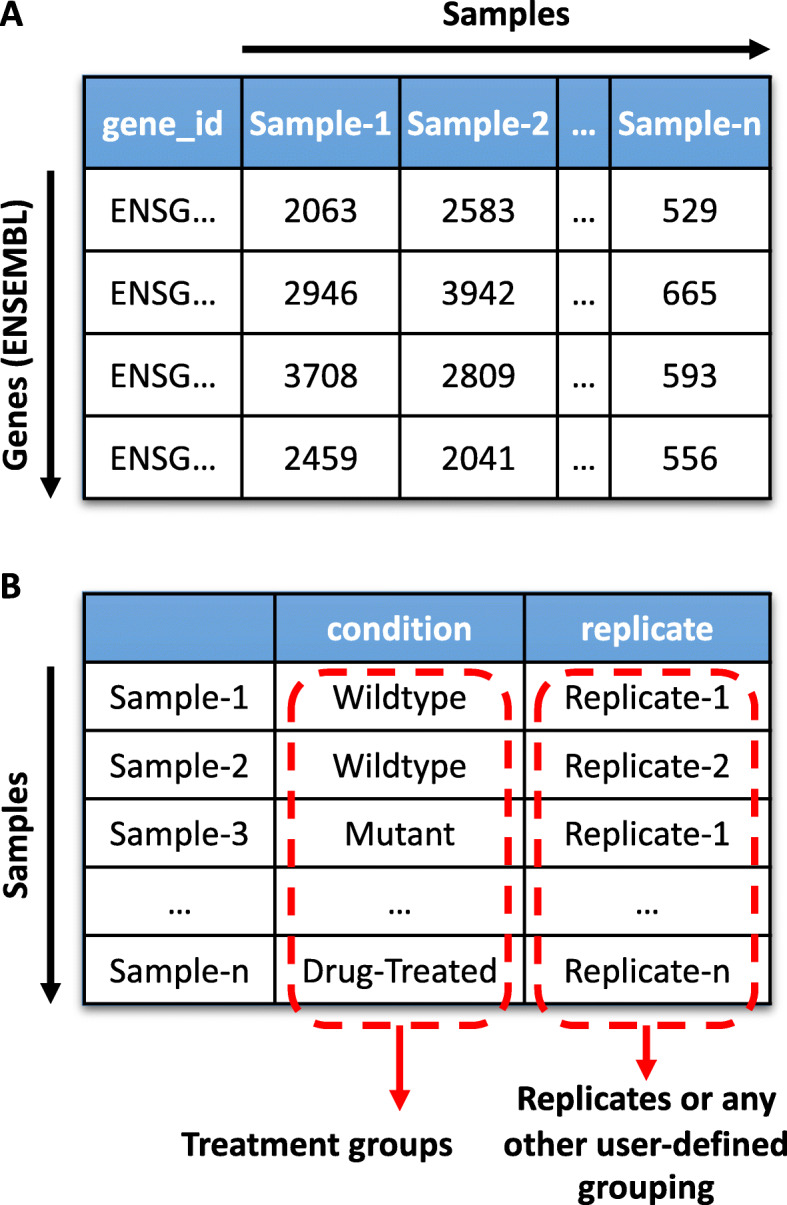
BEAVR requires two inputs: a read count table file and a sample treatment matrix file. **a**BEAVR requires raw, unnormalized read counts as input. This can be obtained using tools such as STAR or HTSeq. The first column of the read count table file must have the heading gene_id and contain unique ENSEMBL IDs. Every column after must contain read counts for one sample, each with a unique identifier in the heading (e.g. Sample-1, Sample-2, …, Sample-n). The read count table file must be either a TXT or CSV format. **b**BEAVR requires an additional file, called a sample treatment matrix file, that contains characteristics about each sample, such as which treatment group the samples belong to. The first column of this file must contain in each row all the samples found in the read count table file (e.g. Sample-1, Sample-2, …, Sample-n) in the same order. The second column must have the heading condition. The third column must have the heading replicate. In the condition column, users must specify which experimental group each sample belongs to (e.g. Wildtype, Mutant, or Drug-Treated). In the replicate column, users can provide any other additional grouping information or replicate information (e.g. Replicate-1, Replicate-2, …, Replicate-n). The sample treatment matrix file must be either a TXT or CSV format

A typical workflow for RNA-seq analysis using BEAVR is shown in Fig. 1b. Briefly, data is loaded into BEAVR, DGE analysis is performed using DESeq2 and the results are visualized in interactive tables, in graphs and other displays. In the Load Data tab, the user must provide a DESeq2 compatible read count table file containing raw, unnormalized read counts (obtained using alignment tools such as STAR or HTSeq) as well as a sample treatment matrix file (created in a text editor or spreadsheet program). The read count table file (either TXT or CSV) should contain the read quantities for all of the samples in the experiment (Fig. 2a). The first column must contain ENSEMBL identifiers for each gene. The heading for this column must be gene_id. The next *n* columns must contain raw read counts for each of the *n* samples. The headings for these *n* columns must be unique sample identifiers (e.g. wildtype-1, wildtype-2, wildtype-3, mutant-1, mutant-2, mutant-3). The sample treatment matrix file (either TXT or CSV) informs BEAVR which columns (samples) in the read count table file belong to which treatment groups (Fig. 2b). This allows multiple replicates to be grouped together across different experimental conditions. The first column must list in each row the sample identifiers for all *n* columns in the read count table file (e.g. wildtype-1, wildtype-2, wildtype-3, mutant-1, mutant-2, mutant-3). The second column of the sample treatment matrix file specifies which experimental condition each sample belongs to (e.g. wildtype and mutant, or untreated and drug-treated). The heading for this column must be condition. In the third column, the user may specify any additional characteristics for each sample, such as replicate numbers/letters or genotype groups (e.g. replicate-A, replicate-B, replicate-C). The heading for this column must be replicate. Both the read count table file and the sample treatment matrix file must contain at least two experimental conditions with a minimum of 2 samples each. Treatment groups do not need to contain the same number of samples in each group.

In the Settings tab, the user must select a control condition and a treatment condition (condition choices are loaded from those available in the sample treatment matrix file). For DGE analyses, DESeq2 is used to compare the selected treatment condition against the selected control condition. The user may specify a minimum cutoff for reads if desired (reads below this cutoff value are dropped before analysis), specify a false discovery rate (FDR) to determine adjusted *p* values (*p*_*adj*_) and also specify an effect size shrinkage method using DESeq2 [8] or apeglm (approximate posterior estimation) [9].

### Representation of results & data exploration

Clicking on the Gene Table tab will initiate automated DGE analysis using the parameters specified by the user. A progress bar will be shown in the bottom right of the main work area. Upon completion, an interactive table displays the results including gene IDs as HUGO Gene Nomenclature Committee (HGNC) symbols, log_2_ fold changes (LFC), *p* values and *p*_*adj*_ values for each gene. Controls in the sidebar may be used to filter the table as desired and a copy may be saved using the Download Table button.

Visualization of all plots is implemented using ggplot2. The PCA tab will generate a principle component analysis (PCA) plot and display all the samples found in the read count table file. In the Sample Clustering tab, the user can select a distance measurement method to use (Pearson correlation, Euclidean, Maximum, Manhattan, Canberra, Binary, or Minkowski) which will compute a distance matrix using the ComplexHeatmap and dist packages and display the sample variation as a heatmap. The Read Count Plots tab will generate normalized read count plots, either as boxplots or jitter plots, for desired genes. The user can enter gene names separated by a comma and change the grid layout as desired (use a 1 × 1 grid for a single plot or increase the grid size as necessary to fit multiple plots). The Heatmap tab will allow the user to generate a heatmap with gene clustering for the top *n* significantly variable genes (where *n* is a user-defined number), or for any list of genes entered by the user. Dependence of the variance on the mean is removed using either variance stabilization (vst) or regularized logarithm (rlog) transformations [8] as specified by the user. The user can also specify a hierarchical clustering method (Ward.D/D2, Single, Complete, Average, McQuitty, Median, or Centroid) to be used by the hclust package (for row and/or column clustering) and a distance measurement method as described above. The Volcano Plot tab will generate a volcano plot using the EnhancedVolcano package to illustrate differentially-expressed genes that meet the user-defined LFC and *p*_*adj*_ cutoffs for the control and treatment conditions specified on the Settings tab. Pathway over-representation analysis and gene set enrichment analysis (GSEA) are performed using the ReatomePA and enrichplot packages [10] and figures are shown in the Pathway Enrichment Plot, Pathway Enrichment Map, GSEA Plot and GSEA Map tabs with the tabular results being displayed in the Pathway Enrichment Table and GSEA Table tabs. All customization options are presented in the sidebar and allow users to control many parameters when plotting figures, including the ability to customize colors, font sizes and legend positions and directions (horizontal or vertical) for all figures. The size and aspect ratio of all figures can be adjusted by clicking and dragging the outside edges of the plot area. The Save Plot button located above every plot allows figures to be saved in multiple formats (JPEG, PDF, PNG, SVG, TIFF) while the Download Table button in the sidebar allows data from any table to be saved (CSV).

### Installation

Since BEAVR is developed in R (+ 3.5), it is OS-independent and runs on Linux, Mac OS and Windows. We provide several methods to install and use BEAVR depending on user preference: 1) the easiest method for those unfamiliar with R is to install Docker (https://docker.com) and use our Docker container (https://hub.docker.com/r/pirunthan/beavr) which comes pre-installed with all of the required components; or 2) users can use our OS-specific scripts to install and configure R with all of the required packages for BEAVR; or 3) users who already have R installed can download BEAVR from GitHub. Additionally, system administrators may install BEAVR in a multi-user server environment which is useful for research groups that want to have a centralized server for BEAVR. This is implemented using ShinyProxy (https://shinyproxy.io) and Docker which provide a secure, sandboxed environment for every connected user. We provide automated install scripts on GitHub to easily accomplish this and system administrators can customize the installation to their specific network requirements. Each of these methods simplify and streamline setup for novice and expert users alike and are well-documented on the GitHub page for BEAVR located at https://github.com/developerpiru/BEAVR.

### Run time consideration

Computation time is dependent on the user’s device specifications since all DGE analyses, statistical tests and visualization steps are performed locally (or the server specifications when running BEAVR on a shared server). For a typical mammalian RNA-seq experiment containing two experimental groups with three replicates each using the human genome as a reference (88 million reads total), automated calculations will take approximately 1 min with a dual-core Intel Core i5 CPU and 4 GB RAM or approximately 30 s with a 6-core Core i7 and 16 GB RAM. Generation of each figure, as well as subsequent modifications thereto, will take a few additional seconds. These short processing times will allow users to repeatedly manipulate experimental settings to recalculate DGE as desired with different parameters. Users may then explore the results, generating figures and filtering and downloading the data for downstream applications.

## Results & discussion

### A typical use case

To demonstrate a typical use case for BEAVR, we utilized a previously published RNA-seq dataset by Sehrawat et al. [11]*.* In this study, LNCaP cell cultures were treated with either DMSO or SP2509 (a small molecule lysine-specific demethylase 1 [LSD1] inhibitor) for 24 h [11]. RNA-seq was performed on RNA harvested from triplicate cell cultures corresponding to each treatment condition. We downloaded raw, unnormalized read counts from GEO (GSE59009) and merged the read counts from all samples to make a single read count table file (TXT). We created a sample treatment matrix file (CSV) using Microsoft Excel to specify the treatment condition group (either DMSO or SP2509) and replicate number for each sample. Once these two files were prepared, they were loaded into BEAVR from the Load data tab. In the Settings tab, we selected ‘DMSO’ as the control condition and ‘SP2509’ as the treatment condition. The FDR was set to 10% and the minimum threshold to drop reads was set to 10.

**Fig. 3:**
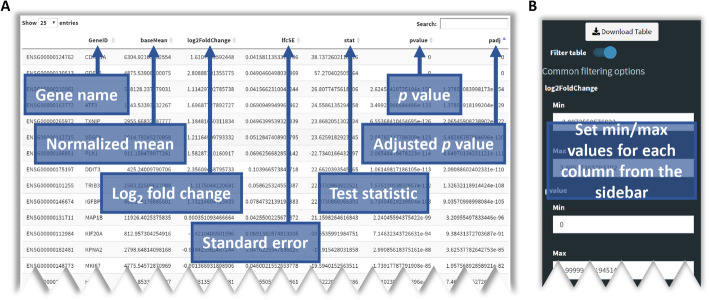
DGE table output from a typical use case for BEAVR. **a** Once DGE analysis completes in BEAVR, an interactive table is shown in the Gene Table tab. This table provides users with log_2_ fold change (LFC) values for each gene as well as *p* values and adjusted *p* values (*p*_*adj*_). Users can search for a particular gene of interest by its gene name or sort the table based on the contents of any column. A copy of the table can be saved using the download button in the sidebar. The data shown here is the output of DGE analysis performed on the Sehrawat et al. dataset. ‘DMSO’ was selected as the control condition and ‘SP2509’ was selected as the treatment condition in the Settings tab. The false discovery rate (FDR) was set to 10% and genes with less than 10 reads were dropped from analysis. **b** The DGE results table in the Gene Table tab can be filtered by any metric using the controls provided in the sidebar. The available filtering options are min/max LFC, min/max *p* value, min/max *p*_*adj*_, min/max baseMean (normalized mean), min/max lfcSE (LFC standard error) and min/max stat (test statistic). The filtered table can be downloaded using the download button in the sidebar. If filtering is enabled, the filtered table will be used to generate the volcano plot in the Volcano Plot tab

Figure 3a shows the DGE results from the Gene table tab, which has been sorted by ascending *p*_*adj*_ values. This table can be saved as-is or it can be filtered. For example, it is often desirable to have a list of only those genes that exceed a specific LFC threshold (e.g. ±1.0) and fall below a *p*_*adj*_ threshold (e.g. < 0.05). These values can be set using the sidebar (Fig. 3b) and the results table will be updated automatically to display genes meeting the selected criteria. These parameters also instruct the thresholds used in generating the volcano plot and pathway analyses.

**Fig. 4:**
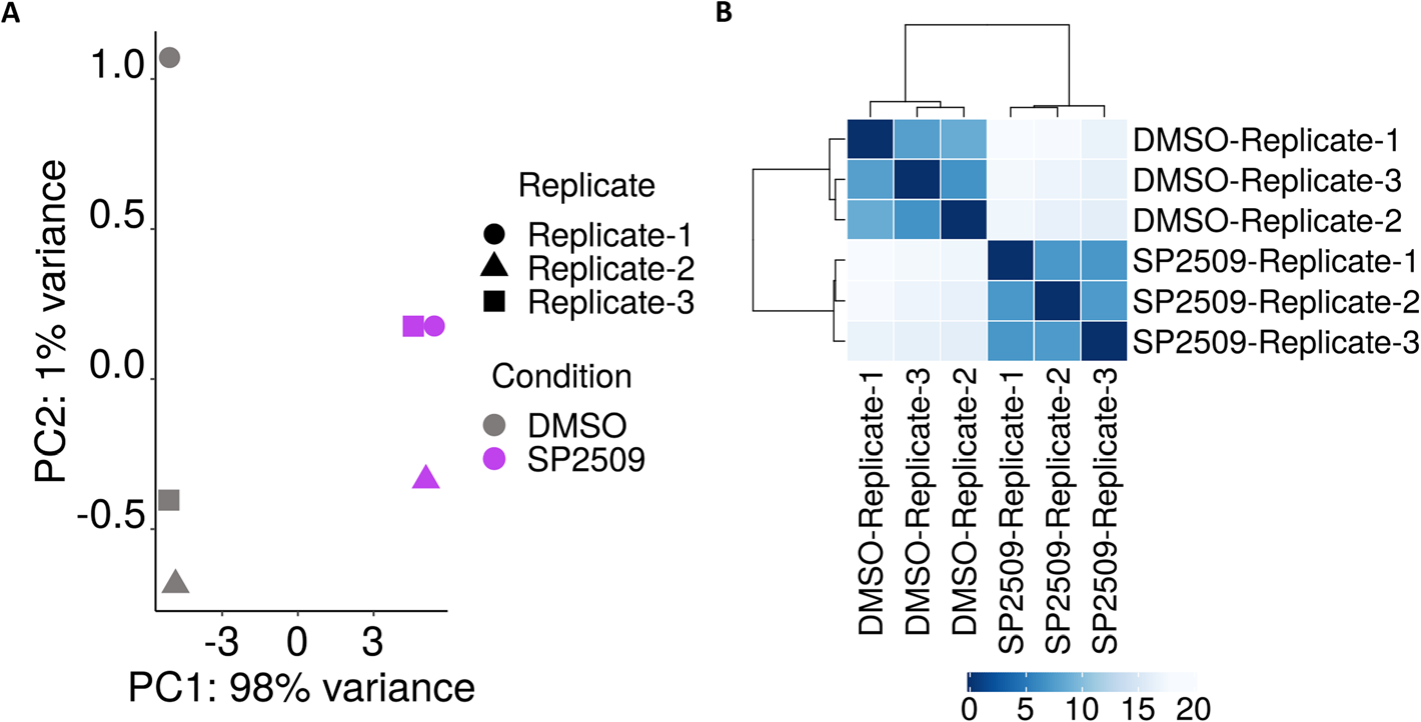
Illustrating variance across samples using principle component analysis (PCA) and sample clustering. **a** PCA is a useful tool to determine the variance within and across different experimental groups and replicates. The PCA output from the PCA tab is shown for the Sehrawat et al. dataset. High variance (98%), as expected, is observed between the two experimental groups (DMSO- vs SP2509-treated) whereas low variance (1%) is observed between replicates within each group. **b** Hierarchical sample clustering is also a useful tool to determine variances. The output from the Sample Clustering tab is shown for the Sehrawat et al. dataset. Pearson correlation was selected as the distance measurement method in the sidebar. Similar to the PCA plot, the clustered heatmap shows that replicates in each experimental group (DMSO- or SP2509-treated) cluster strongly together, indicating low variance between biological replicates

PCA is an important consideration in RNA-seq analysis for small and large studies. Depending on the experimental design, PCA plots can be used for quality control or as a discovery tool [12]. In studies with only two control groups and just two or three biological replicates, it can inform researchers of replicates that are not congruent and have high variance which can skew results and reduce statistical power. In larger studies, it can provide insight into the heterogeneity within experimental conditions. The PCA Plot tab displays a PCA plot from our example dataset. The plot shows that there is a very small amount of variance (1%) between replicates within each experimental group (DMSO- or SP2509-treated), while there is very large variance, as expected, between the two experimental groups (98%) (Fig. 4a). Further quality control and insight into sample and replicate variation can be interrogated through a distance matrix and subsequent sample clustering. We defined the parameters in the Sample Clustering tab to compute Pearson correlation distances and the result is shown in Fig. 4b. Replicates in the same experimental group cluster together and are very similar to each other, indicating very low variance. Together, these two graphs provide researchers with useful information about experimental groups and consistency of biological replicates.

**Fig. 5:**
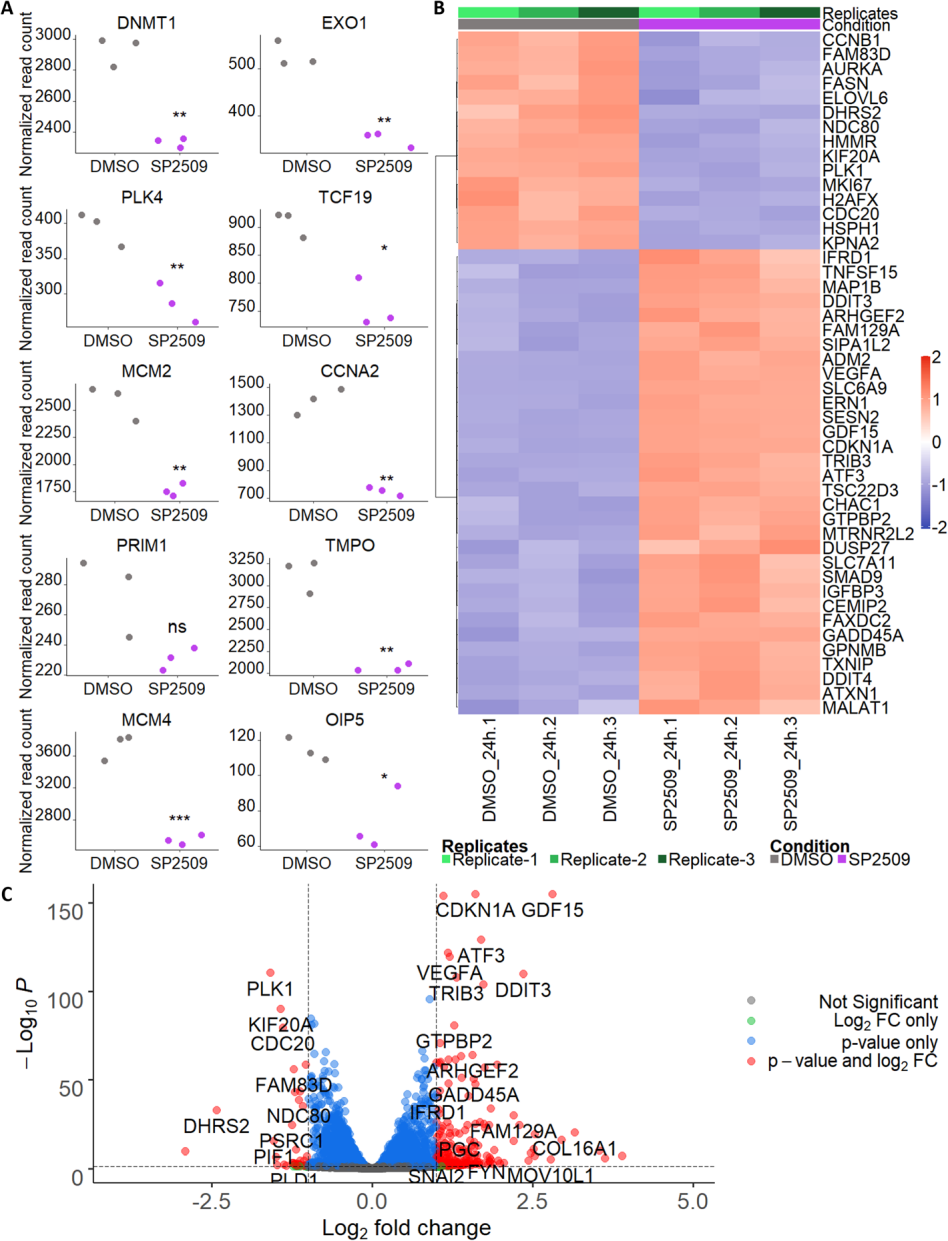
Visualizing normalized read counts and differential gene expression between experimental groups. **a** Normalized read count plots are shown for ten embryonic stem cell-like genes of interest from the Sehrawat et al. dataset to illustrate changes between the DMSO- and SP2509-treated groups. BEAVR allows users to enter a list of genes to illustrate expression behavior as jitter plots (shown) or boxplots (not shown). A 5 × 2 (rows x columns) grid was selected to display these 10 genes. **b** The top 50 most differentially-expressed genes between DMSO- and SP2509-treated groups are shown in the clustered heatmap for the Sehrawat et al. dataset. This heatmap was generated in the Heatmap tab using the Ward.D2 hierarchical clustering method and Euclidean distance measurements. Row (gene) clustering was enabled. Clustered heatmaps are useful for displaying expression changes across treatment groups. **c** A volcano plot highlighting genes that meet both LFC and *p*_*adj*_ cutoffs are shown for the Sehrawat et al. dataset. This volcano plot was generated in the Volcano Plot tab with the LFC cutoff set to ±1 and the *p*_*adj*_ cutoff set to < 0.05. The volcano plot is another way to visualize the data shown in the heatmap in (**b**), however the volcano plot also illustrates the statistical significance of genes (the *y*-axis)

Sehrawat et al. found inhibition of LSD1 in LNCaP cells caused downregulation of previously characterized embryonic stem cell-like genes [11, 13]. Using the Read Count Plots tab, we explored the normalized read counts of these genes and generated plots that showed reduced normalized reads in the SP2509-treated cells compared to DMSO-treated cells (Fig. 5a). In situations where genes or pathways of interest are already known, read count plots can be used as a tool to investigate changes in gene expression across samples. However, RNA-seq is also used in experimental systems to inform researchers of genes and pathways that may be of interest. For such purposes, a heatmap with gene clustering or a volcano plot are useful tools. The Heatmap tab generates heatmaps for the top *n* genes (where *n* is a user-defined number) or for specific genes entered by the user. Figure 5b shows the top 50 most differentially-expressed genes after variance stabilization with hierarchical clustering performed across rows (Ward.D2 method). This provides information on the most strongly upregulated and downregulated genes. Although the data for a heatmap is transformed and variance is stabilized, it does not provide information on significance (*p* values or *p*_*adj*_) [8]. The volcano plot from the Volcano Plot tab illustrates genes that meet a specified LFC threshold as well as a *p*_*adj*_ threshold (Fig. 5c). We set the LFC threshold to ±1.0 and the *p*_*adj*_ cutoff to < 0.05. Genes highlighted in red (meeting both the LFC and *p*_*adj*_ cutoffs) were also found in the heatmap, demonstrating the usefulness of heatmaps and volcano plots and how the two can be used together for discovery of novel gene expression patterns.

**Fig. 6:**
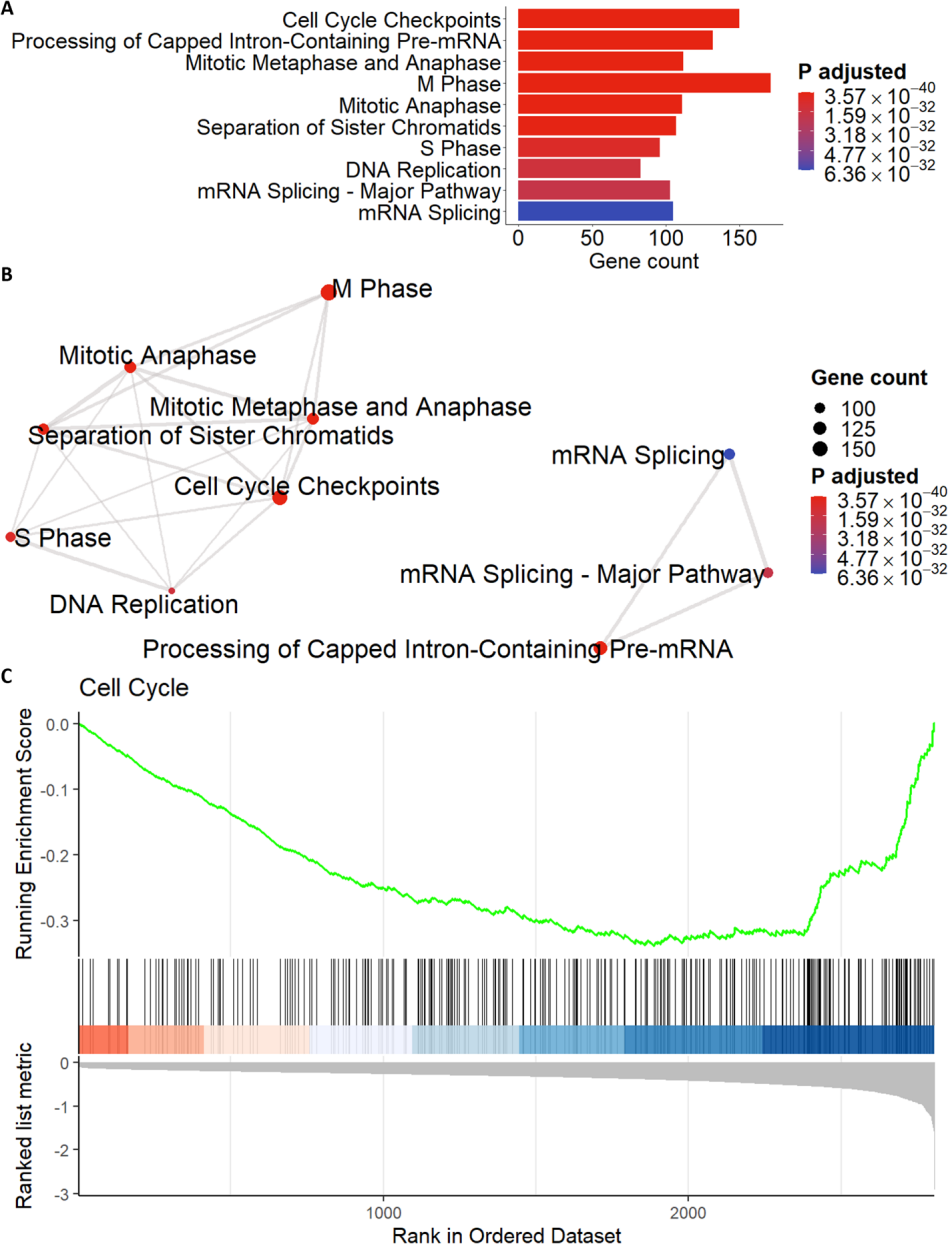
Identification of enriched pathways among differentially expressed genes. **a** Bar graph showing the results of over-representation analysis using the Pathway Enrichment Plot tab. The maximum number of pathways/categories to show was set to 10 and the enrichment *p*_*adj*_ value cutoff was set to < 1 × 10^− 30^. The gene count (*x-*axis) indicates the number of genes enriched in each pathway and colors indicate level of significance (*p*_*adj*_). The pathways are plotted on the *y*-axis in order of increasing significance. **b** While the Pathway Enrichment Plot tab shows a bar graph or dot plot for only a subset of enriched pathways, the Pathway Enrichment Map tab shows all of the enriched pathways in an interconnected network map. The size of each node indicates the gene count (number of genes enriched in each category) and the color represents the *p*_*adj*_ value (the cutoff was set to < 1 × 10^− 30^). **c** The GSEA Plot tab generates a plot of the running enrichment score for a specified pathway/category. The plot for the category “cell cycle” is illustrated here. Currently only Reactome pathways/categories are supported for each of these figures. The input data for **a**-**c** is the filtered or unfiltered data from the Gene Table tab (we set the LFC to < 0 and the *p*_*adj*_ cutoff to < 0.05)

Following identification of upregulated and downregulated genes, it is useful to perform pathway enrichment or gene set enrichment analysis (GSEA) [14, 15] to identify important pathways of interest that will inform investigators of downstream experiments. The Pathway Enrichment Plot tab performs over-representation analysis and produces either a dot plot or bar graph of the top *n* pathways (where *n* is a user-defined number) (Fig. 6a). The Pathway Enrichment Map tab provides a broader look at all enriched pathways using an interconnected network map (Fig. 6b) that shows the results of over-representation analysis, however users may also wish to perform GSEA on the GSEA Map tab. The GSEA Plot tab displays a plot of the running enrichment score for a specific enriched pathway as defined by the user (Fig. 6c). The input data used to generate these figures is the filtered or unfiltered data from the Gene Table tab (we filtered the data using LFC < 0 and *p*_*adj*_ < 0.05). The pathways identified in Fig. 6a-c are consistent with the most downregulated genes shown in the heatmap (Fig. 5b) and volcano plot (Fig. 5c) (such as *H2AX, CDC20, CCNB1, AURKA*) and indicate the most significantly enriched pathways among downregulated genes are related to cell cycle and DNA replication processes. Together, the read count plots, heatmap, volcano plot and pathway plots inform researchers of gene expression changes and provide insight into which genes and pathways may play an important role in their experimental system.

### Future work

DGE analyses computes differences between two groups at a time, such as Wildtype and Single-knockout, even though users can load data files containing > 2 groups (e.g. Wildtype, Single-knockout and Double-knockout). Currently, users must perform one comparison first (e.g. Wildtype vs Single-knockout), download the results and then perform another comparison (e.g. Wildtype vs Double-knockout) and download the new results. Users must then manually perform comparisons outside of BEAVR to identify overlapping or non-overlapping genes. Future updates to BEAVR will allow users to perform multiple DGE analyses and allow them to interact with both results at once to perform direct comparisons within BEAVR. Implementation of additional plotting tools, such as Euler or Venn diagrams, will allow for the visualization of overlapping or non-overlapping dysregulated genes across different comparisons such as Wildtype vs Single-knockout and Wildtype vs Double-knockout. These overlapping or non-overlapping datasets can then be used to perform pathway analysis or GSEA within BEAVR.

Presently, BEAVR only supports Reactome categories for pathway analysis and GSEA. Future updates will enable support for Gene Ontology (GO) [16], Disease Ontology (DO) [17], KEGG [18], WikiPathways [19] and Molecular Signature Database (MSigDb) [14, 20] to provide users with more options.

## Conclusions

RNA-seq analyses has largely relied on command-line-driven tools, such as DESeq2 [8], EdgeR [21] or ALDEx [22], thereby creating a barrier to entry for scientists wishing to conduct RNA-seq analyses. Here we presented BEAVR, a graphically-driven tool that greatly simplifies DGE analyses through a logical workflow that makes use of DESeq2 as the core DGE engine. BEAVR is easy-to-use and allows researchers to not only quickly and easily change experimental parameters in real-time to visualize results, but also provides an intuitive interface for researchers to explore their results in-depth and generate highly customizable figures. Various other tools have been developed to provide users with graphical interfaces for RNA-seq analyses, most notably GENAVi [23], START [24], iDEP [25], DEBrowser [26], DEIVA [27] and DEApp [28]. While these tools have undoubtedly provided a significant evolution in RNA-seq analysis tools, we found that BEAVR offers meaningful advantages in comparison. Specifically, the ease of installation and usage, combined with more flexibility in data output features are important advancements. None of these programs offers each of our key features in one complete package, such as filtering capabilities of gene lists, all of the different data displays that BEAVR provides (heat-map, PCA plots, etc.), the ability to customize and export figures in as many formats, or the ability to integrate pathway analysis. Based on these differences we expect BEAVR will be widely utilized.

BEAVR was developed to be simple enough for novices, yet fast and powerful enough for experts to streamline and automate DGE analyses. Even with modest computing power by today’s standards, BEAVR is capable of completing analyses within minutes, allowing researchers to quickly automate analyses of large datasets. With uses for RNA-seq continuing to expand — both experimentally and clinically — BEAVR is well-positioned to allow analysis of these datasets to be quick and efficient, while providing the latitude for customization as per the user’s requirements.

## Availability and requirements

**Project name:** BEAVR

**Project home page:**https://github.com/developerpiru/BEAVR and https://hub.docker.com/r/pirunthan/beavr

**Project documentation:** https://github.com/developerpiru/BEAVR/blob/master/README.md

**Operating system:** Linux, Mac OS, Windows

**Programming language:** R

**Other requirements:** R 3.5 or higher, web browser

**License:** GNU General Public License v3.0

**Any restrictions to use by non-academics:** None

## Data Availability

The dataset used in this article is available in the GEO repository, (GSE59009).

## Abbreviations

BEAVR: Browser-based exploration and visualization of RNA-seq data
DGE: Differential gene expression
DO: Disease Ontology
FDR: False discovery rate
GO: Gene Ontology
GSEA: Gene set enrichment analysis
GUI: Graphical user interface
HGNC: HUGO Gene Nomenclature Committee
KEGG: Kyoto Encyclopedia of Genes and Genomes
LFC: Log_2_ fold change
LSD1: Lysine-specific demethylase 1
MSigDb: Molecular Signature Database
OS: Operating system
*p*_*adj*_: Adjusted *p* value
PCA: Principle component analysis
RNA-seq: RNA-sequencing
